# An interpretable vibration-enhanced BRB model for rolling bearing fault diagnosis

**DOI:** 10.1371/journal.pone.0342757

**Published:** 2026-03-13

**Authors:** Ziyu Fan, Kangle Li, Hailong Zhu, Cuiping Yang, Wei He

**Affiliations:** 1 School of Computer Science and Information Engineering, Harbin Normal University, Harbin, China; 2 School of Computer Science and Mathematics, Harbin Finance University, Harbin, China; 3 Graduate School, Harbin Normal University, Harbin, China; Universiti Teknologi Malaysia, MALAYSIA

## Abstract

The operational condition of rolling bearings is essential to the reliability of industrial machinery, making fault diagnosis a critical research topic. Although deep learning has gained widespread attention in this domain, its black-box character and reliance on a large number of training samples limit its practical applicability. In contrast, knowledge-driven intelligent diagnostic models have gained increasing interest due to their superior interpretability and robustness under small-sample conditions. The Belief Rule Base (BRB) model is a representative example of such interpretable methods. However, the conventional BRB models struggle to process continuous signals, limiting their effectiveness in real-world bearing fault diagnosis. This study proposes a novel Vibration-Enhanced Belief Rule Base (VE-BRB) model designed to address this limitation. First, the window feature extraction method is used to preprocess the continuous vibration signal. Bearing fault features are extracted from low-frequency and high-frequency to construct an energy matrix representation. Thereby, the original continuous vibration signal can be effectively mapped to the rule matching space. Second, the model is reasoned through an evidential reasoning (ER) algorithm. This guarantees the interpretability of the model. Finally, the projection covariance matrix adaptation evolution strategy (P-CMA-ES) is employed as the optimization process. To validate the effectiveness of the proposed method, the test was performed using bearing datasets from Case Western Reserve University and Huazhong University of Science and Technology under various conditions.

## 1. Introduction

Bearings are key components of all types of rotating machinery and are widely used in electric motors, wind turbines, train wheels, and other equipment. Bearings are often subjected to excessive loads, insufficient lubrication, and contaminant ingress during operations. These factors can cause surface wear, fatigue cracking, and vibration abnormalities. Therefore, bearing fault diagnosis [1] has become an essential and valuable research field.

As critical components of complex mechanical systems, bearings play a decisive role in maintaining operational performance and safety. In safety-critical domains such as aerospace, transportation, and power generation, bearing failures can trigger system malfunctions. Accordingly, fault diagnosis must not only ensure high accuracy but also provide interpretability [2], ensuring that diagnostic decisions are transparent and trustworthy. In current research, fault diagnosis models can be broadly classified into three categories: black-box models, white-box models, and gray-box models.

Black-box models are built entirely from observed data rather than a priori knowledge [3]. Deep learning models rely on abstract features and large amounts of labeled data, which obscure the reasoning process and limit interpretability in safety-critical applications. In recent years, deep learning has become the dominant method among black-box models for bearing fault diagnosis. Convolutional neural networks (CNNs) [4,5] have been extensively employed to extract spatial and spectral features from vibration signals, while recurrent neural networks (RNNs) [6] and long short-term memory (LSTM) [7] are commonly used to capture temporal dependencies in sequential data. Owing to black-box models’ lack of interpretability, engineers often hesitate to fully trust their results [8].

White-box models are established according to system principles [9]. Wang utilized fault tree analysis to identify the errors and causes of critical components, providing a fully transparent reasoning structure based on logical dependencies [10]. However, as the complexity of real-world systems increases, analyzing system principles becomes increasingly challenging. Consequently, white-box modeling methods have become less practical.

To solve these problems, gray-box models have been developed by combining prior knowledge with observational data [11]. The common gray-box model with IF-THEN rules has an understandable semantic description and a clear reasoning process [12,13]. Soni employed a fuzzy logic controller with fuzzy clustering, model combining fuzzy logic reasoning with data-driven fuzzy clustering, enabling interpretable transformer fault analysis [14]. And, Feng et al. developed a gray-box diagnostic framework based on multi-source heterogeneous data fusion [15].

Under the above black-box, white-box, and gray-box modeling paradigms, interpretability is achieved through different mechanisms. To mitigate the limited interpretability of black-box models, various explainable deep learning methods have been developed [16]. For example, Zhou et al. proposed a rolling bearing fault diagnosis method based on a convolutional neural network with a frequency attention mechanism, which enhances the model’s ability to focus on fault-related spectral components [17]. However, such methods typically provide post-diagnostic explanations rather than intrinsic transparency, and the reliability of their interpretations remains difficult to verify. White-box signal-processing-based methods, such as the time–frequency ridge estimation approach proposed by Li et al. for gear and bearing fault diagnosis under time-varying speeds [18], explicitly extract dominant frequency trajectories from time–frequency representations to provide physically interpretable fault-related features. Nevertheless, their role is largely limited to feature analysis, as diagnostic conclusions typically depend on expert interpretation rather than an explicit decision-making mechanism. In contrast, gray-box rule-based methods emphasize decision-level interpretability by incorporating explicit diagnostic rules and inference procedures, enabling diagnostic conclusions to be derived in a transparent and traceable manner. Among gray-box models that emphasize decision-level interpretability, the Belief Rule Base (BRB) provides a transparent rule-based reasoning framework.

Belief Rule Base (BRB) is a typical gray-box modeling method [19]. It applies expert knowledge and experience and provides a preliminary qualitative analysis of the system. When dealing with data situations with uncertainty and ambiguity, BRB provides diagnostic results that guarantee the rationality of system analysis and interpretability. For safety-critical equipment, maintenance engineers must not only determine whether a fault exists but also comprehend the underlying physical reasoning behind the diagnosis to formulate appropriate corrective actions. A transparent reasoning mechanism enables engineers to validate decision-making processes against measurable vibration responses and accumulated domain expertise [20]. However, because the design of BRB models is based on discretized rules [21], continuous input signals may be completely meaningless when transformed into rule bases. This affected the diagnostic accuracy and adaptability of the model.

Therefore, to address the above issues, this study proposes a novel Vibration-Enhanced Belief Rule Base (VE-BRB) model for rolling bearing fault diagnosis, designed to retain interpretability while adapting to the characteristics of vibration signals. Firstly, a window-based discretization mechanism is utilized. This mechanism divides the continuous time-domain signal into a series of short-time segments [22], within which the statistical features are extracted and represented as discrete antecedent attributes. Such a design enables the BRB to directly process vibration data while maintaining the temporal locality of fault-related patterns. Subsequently, a dual-frequency feature extraction mechanism is employed [23]. It extracts fault-related features from the low- and high-frequency components of the vibration signal. In bearing fault diagnosis, the low-frequency features mainly reflect cyclic variations correlated with different fault types [24], while the high-frequency components are more reflective of localized defects. High-frequency and low-frequency features are considered different input attributes. Their relative influence can be dynamically adjusted by attribute weights in the Evidential Reasoning (ER) algorithm [25,26].

Through the above improvements, the VE-BRB model demonstrates several significant advantages in bearing fault diagnosis. In contrast to interpretable black-box and white-box models, the inference process of VE-BRB is governed by explicit rule activations and evidential fusion, allowing each diagnostic decision to be decomposed into traceable contributions from the corresponding rules and vibration-signal components. Moreover, the dual-frequency components (low- and high-frequency bands) involved in the inference correspond to carrying distinct physical meanings [24,27]. Each rule is driven by observable frequency-domain evidence, enabling the belief fusion process to preserve the causal relationship between spectral characteristics and diagnostic conclusions. This mechanism grounds the inference in measurable vibration responses rather than abstract statistical correlations, allowing engineers to identify the physical root causes of faults. This dual-frequency representation also mitigates the influence of noise by isolating information in both low- and high-frequency bands. Therefore, the proposed method is particularly suitable for safety-critical fault diagnosis scenarios or industrial environments that demand decision traceability and interpretability.

The innovations of VE-BRB primarily lie in the following aspects:

1. Interpretable bearing diagnosis via BRB: This study utilizes the BRB model’s advantages in interpretability for bearing-fault diagnosis. By integrating rule-based reasoning with a transparent evidential fusion process, the model ensures that diagnostic decisions remain traceable.
2. Discretize continuous inputs to achieve compatibility with BRB inputs: A window-based feature extraction mechanism converts continuous vibration signals into sequences of discrete feature segments compatible with BRB, thereby enabling the BRB to process vibration data effectively.
3. Dual-frequency feature energy representation: In addition to the discretization, dual-frequency features are extracted from vibration signals to build the energy matrix input. This design attenuates noise while retaining fault-salient characteristics, ensuring interpretable decisions and rule traceability throughout the diagnostic process.

The remainder of this paper is organized as follows: Section 2 discusses the primary challenges in vibration-based fault diagnosis. Section 3 provides a detailed description of the theoretical foundations of the proposed model. Section 4 reports experiments verifying the effectiveness and interpretability of the VE-BRB model. Section 5 concludes the paper.

## 2. Problem description and solution framework

The problems are formulated in II.A, and the diagnostic procedure of the VE-BRB is elucidated in II.B.

### 2.1 Problem formulation

Problem 1: Representation of Continuous Vibration Signals in BRB

In vibration-based fault diagnosis, the measured vibration signal s(t) is continuous in time, whereas the BRB model operates on discrete antecedent attributes. To bridge this mismatch, a windowing operation is applied to segment the continuous sequence into multiple short-time intervals, each treated as an independent observation for subsequent reasoning.

Let w(·) denote the window function of length P. The signal within the $z_{th}$ window is expressed as


$$s_{k}\left(t\right)=s\left(t\right)\cdot w(t-zP),\;z=1,2,\text{\textbackslash{}ldots,}M$$ (1)

where M is the total number of windows. Each $s_{k}\left(t\right)$ retains the local temporal characteristics of the original signal within its interval.

A feature extraction operator F(·) is then applied to obtain representative descriptors:


$$x^{\prime }=F\left(s_{z}\left(t\right)\right)$$ (2)

The resulting discrete feature set $x^{\prime }$ forms the input information for the BRB model.

Problem 2: Dual-Frequency Energy Representation for Interpretable Inference

However, although the window-based representation discretizes the continuous vibration signal, it remains uncertain how specific spectral components influence rule activation within the BRB.

This limitation motivated the design of a dual-frequency representation, in which the vibration information within each window is decomposed into complementary components that carry distinct physical meanings.

Accordingly, each windowed signal sz(t) is decomposed by a discrete wavelet transform 𝒟(·) into its approximation and detail components, denoted as cA and cD respectively. These coefficients correspond to the relatively low- and high-frequency bands at the current decomposition level, reflecting the global and local behaviors of the vibration signal:


$$\begin{array}{c} 𝒟\left(s_{z}\left(t\right)\right)=\left[s_{z}^{\text{low}}\left(t\right),s_{z}^{\text{high}}\left(t\right)\right]\text{\textbackslash{}hspace\{1em}\}\Rightarrow \text{\textbackslash{}hspace\{1em}\}cA,cD \end{array}$$ (3)

The corresponding energies of the two components are computed as


$$E_{A}=\sum (cA{)}^{\text{2}},\text{\textbackslash{}hspace\{1em}\}E_{D}=\sum (cD{)}^{\text{2}}$$ (4)

The energy pair $\left[E_{A},E_{D}\right]$ forms the dual evidential input for the VE-BRB model. Here, $E_{A}$ primarily represents the structural or cyclic behavior of the bearing, whereas $E_{D}$ captures transient impulses caused by localized defects.

**Fig 1:**
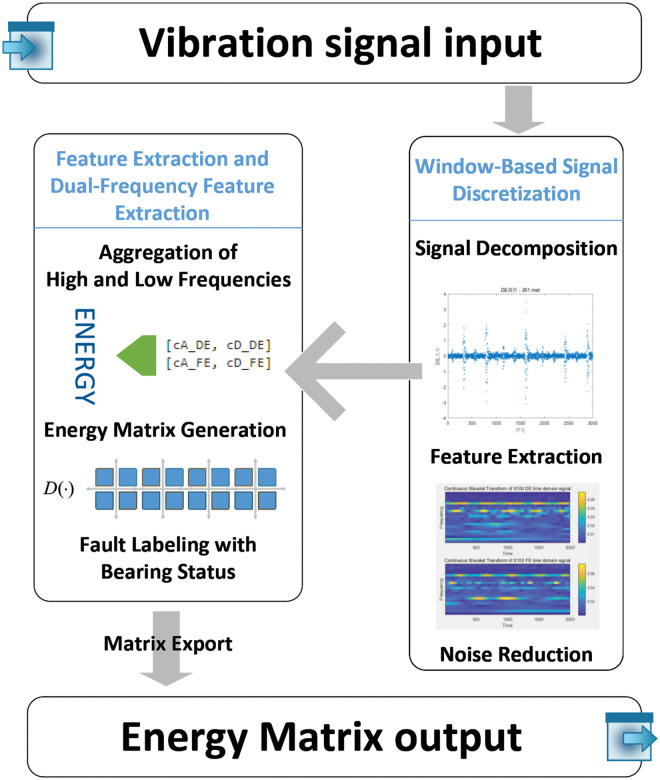
Dual-frequency feature extraction and energy matrix construction process for the VE-BRB model.

The energy matrix including these two values is directly fed into the BRB model for inference and decision-making. This process is illustrated in Fig 1.

### 2.2 Diagnostic procedure of the VE-BRB

Based on the dual-frequency energy matrix constructed in II.A, the diagnostic procedure of the VE-BRB model is designed to transform these interpretable vibration features into rule activations and belief outputs.

The diagnostic workflow of the VE-BRB model is organized as a sequence of interpretable reasoning steps. The main procedures are summarized as follows:

Step 1: Feature representation.

The dual-frequency energy matrix constructed in II.A is used as the input of the BRB model. It establishes the mapping between vibration information and the antecedent attributes of the rule base.

Step 2: Rule Activation and Evidential Fusion.

The VE-BRB performs reasoning through rule matching, activation, and evidential fusion in III.A. The input features are evaluated against the predefined rules to determine activation strengths, which are then fused by the ER algorithm to produce diagnostic beliefs.

Step 3: Parameter optimization.

The parameters of the VE-BRB are optimized via the Projection Covariance Matrix Adaptation Evolution Strategy (P-CMA-ES) [13,28] in III.B.

Step 4: Diagnostic output generation.

**Fig 2:**
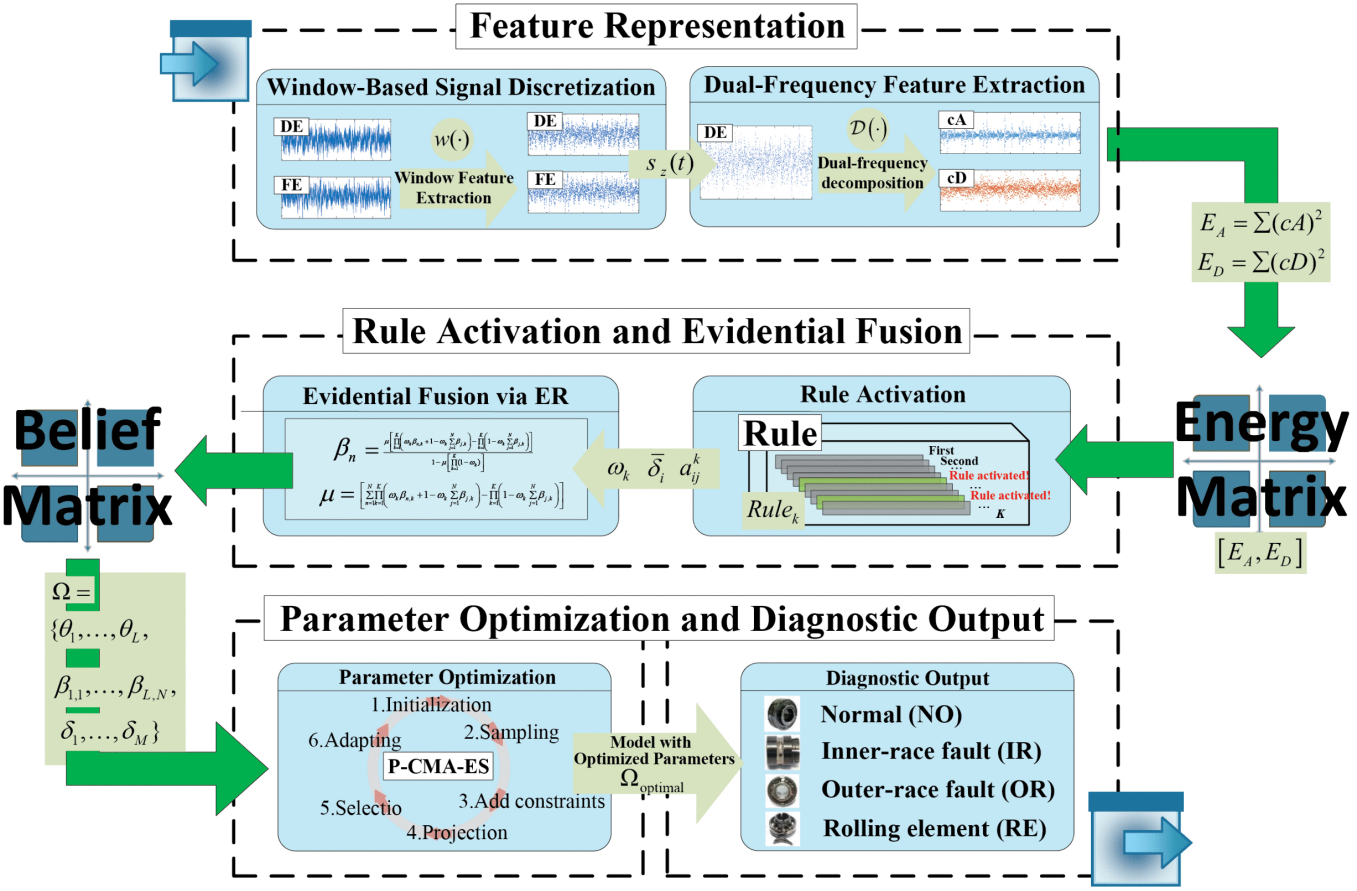
The diagnosis process of the VE-BRB model.

The final diagnosis is made via the optimized model fusion belief degrees. The diagnosis process of the VE-BRB model is illustrated in Fig 2.

## 3. Theoretical foundations of the VE-BRB model

This section presents the theoretical foundations of the proposed VE-BRB model, including its inference mechanism, evidential fusion process, and parameter optimization strategy.

### 3.1 BRB system

The BRB model is composed of a set of belief rules [29]. Consider a dataset DS with n samples and M features $DS=\{\left(\begin{array}{c} @c@x_{i},y_{i} \end{array}\right):i=1...n,x_{i}\in {\mathbb{R}}^{M},y_{i}\in \mathbb{R}\}$. The $k_{th}$ rule is profiled as follows:


$$\begin{array}{c} Rule_{k}:If\;x_{\text{1}}\;is\;A_{\text{1}}^{k}\;and\;x_{\text{2}}\;is\;A_{\text{2}}^{k}\;and\;L\;and\;x_{M}\;is\;A_{M}^{k},\;\\ \;Then\;y\;is\;\left\{\left(D_{\text{1}},\beta_{1,k}),......,(D_{L},\beta_{L,k}\right),\left(D,\beta_{B,k}\right)\right\}\\ \;k\in \{1,2,...,K\}\\ \;with\;rule\;weight\;\theta_{k},attribute\;weight\;\delta_{\text{1}},\delta_{\text{2}},\ldots ,\delta_{M} \end{array}$$ (5)

where $x_{\text{1}},x_{\text{2}},\ldots ,x_{N}$ represent the discretized input; $A_{\text{1}}^{k},A_{\text{2}}^{k},\ldots ,A_{N}^{k}$ denote the reference values of attributes in the $k_{th}$ rule; and $B_{\text{1}},B_{\text{2}},......,B_{L}$ represent the diagnostic conclusions of the rule. $\theta_{k}$ denotes the rule weight of the $k_{th}$ rule. $\delta_{\text{1}},\delta_{\text{2}},\ldots ,\delta_{M}$ are the attribute weight. M is the number of characteristics and L is the number of diagnostic conclusions.

The input samples are converted into belief distributions, and the activation and attribute weights are determined as follows.


$$a_{ij}^{k}=\left\{ \begin{array}{c} @c@\frac{A_{i,l+1}^{k}-attr_{i}}{A_{i,l+1}^{k}-A_{i,l}^{k}},j=l\left(A_{i,l}^{k}\;⩽\;attr_{i}\;⩽\;A_{i,l+1}^{k}\right),\\ \frac{attr_{i}-A_{i,l}^{k}}{A_{i,l+1}^{k}-A_{i,l}^{k}},j=l+1\left(A_{i,l}^{k}\;⩽\;attr_{i}\;⩽\;A_{i,l+1}^{k}\right),\\ 0,else \end{array}\right.$$ (6)

$${\overset{―}{\delta }}_{i}=\frac{\delta_{i}}{\underset{j=1,2...T_{k}}{max}\left\{\delta_{j}\right\}}$$ (7)

$$\omega_{k}=\frac{\theta_{k}\prod_{i=1}^{T_{k}}\;\;{\left(\alpha_{i,j}^{k}\right)}^{\overset{―}{\delta_{i}}}}{\sum_{l=1}^{K}\left[\theta_{l}\prod_{i=1}^{T_{l}}\;\;{\left(\alpha_{i,j}^{l}\right)}^{\overset{―}{\delta_{i}}}\right]}$$ (8)

where $a_{ij}^{k}$ represents the matching degree between the $i_{th}$ attribute of the input sample and its $j_{th}$ reference value $A_{ij}^{k}$ in the $k_{th}$ rule, and $x_{i}$ denotes the input value of the $i_{th}$ attribute. $attr_{i}$ indicates the value of the $i_{th}$ attribute, ${\overset{―}{\delta }}_{i}$ denotes the normalized attribute weight, and $\omega_{k}$ is the activation weight of the $k_{th}$ rule. A rule is considered activated when $\omega_{k}\neq 0$.

The inference of activated belief rules is the analytical ER algorithm described below:


$$\beta_{n}=\frac{\mu \left[\prod_{k=1}^{K}\;\;\left(\omega_{k}\beta_{n,k}+1-\omega_{k}\sum_{j=1}^{N}\beta_{j,k}\right)-\prod_{k=1}^{K}\;\;\left(1-\omega_{k}\sum_{j=1}^{N}\beta_{j,k}\right)\right]}{1-\mu \left[\prod_{k=1}^{K}(1-\omega_{k})\right]}$$ (9)

$$\mu =\left[\sum_{n=1}^{N}\prod_{k=1}^{K}\;\;\left(\omega_{k}\beta_{n,k}+1-\omega_{k}\sum_{j=1}^{N}\beta_{j,k}\right)-\prod_{k=1}^{K}\;\;\left(1-\omega_{k}\sum_{j=1}^{N}\beta_{j,k}\right)\right]$$ (10)

$$Predict=\sum_{i=1}^{N}u_{i}\beta_{i}$$ (11)

where $\beta_{\text{1}},\beta_{\text{2}},...,\beta_{N}$ represent the belief degrees associated with the corresponding consequents $D_{\text{1}},D_{\text{2}},\ldots ,D_{N}$. The variable μ is an intermediate term used for normalization. Equation (11) represents the utility function, while $u_{i}$ indicates the reference value at the $i_{th}$ diagnostic results.

### 3.2 P-CMA-ES optimization strategy

Compared with gradient-based algorithms (SGD, Adam) and swarm intelligence methods (PSO, GA), the P-CMA-ES [30,31] does not rely on explicit gradient information. Moreover, P-CMA-ES can generate solutions strictly within the feasible region, ensuring that all equality constraints are consistently satisfied during the optimization process. The details of P-CMA-ES can be described as follows:

Let the initial parameter vector be $\Omega^{\text{0}}$ represents the initial population mean.


$$\Omega^{\text{0}}=\{\theta_{\text{1}},\ldots ,\theta_{L},\beta_{1,1},\ldots ,\beta_{L,N},\delta_{\text{1}},\ldots ,\delta_{M}\}$$ (12)

During the sampling phase, a new population is generated based on the mean and covariance of the current generation. The sampling operation is performed as


$$\Omega_{i}^{g+1}\sim w^{g}+\epsilon 𝒩\left(0,C^{g}\right),\text{\textbackslash{}hspace\{1em}\}i=1,\ldots ,\lambda ,$$ (13)

where $\Omega_{i}^{g+1}$ denotes the $i_{th}$ solution in the $(g+1{)}_{th}$ generation, $w^{g}$ is the mean of the population, ϵ is the step size, 𝒩 represents the normal distribution, and $C^{g}$ denotes the covariance matrix in generation g.


$$\begin{array}{cc} \Omega_{i}^{g+1}(1+n_{e}\times (j-1) & :n_{e}\times j)=\Omega_{i}^{g+1}(1+n_{e}\times (j-1)\\ \; & :n_{e}\times j)-A_{e}^{T}\times (A_{e}\times A_{e}^{T}{)}^{-1}\times \Omega_{i}^{g+1}(1+n_{e}\times (j-1)\\ \; & :n_{e}\times j)\times A_{e} \end{array}$$ (14)

where $n_{e}$ denotes the number of variables involved in the equality constraint, and $A_{e}=[1\ldots 1{]}_{1\times N}$ defines the parameter vector of the constraint.

This projection step ensures that all generated solutions remain within the feasible region and strictly satisfy equality constraints.

After projection, the selection operation updates the population mean according to


$$w^{g+1}=\sum_{i=1}^{\tau }h_{i}\Omega_{i:\lambda }^{g+1},$$ (15)

The execution of the selection operation to update the mean by Equation (15) denotes the $i_{th}$ solution from λ solutions in the $g+{\text{1}}_{th}$ generation. τ is the offspring population size.

Execute the adapting operation to update the covariance matrix. The above optimization process is iteratively repeated until convergence is achieved, and the best solution $\Omega_{\text{Optimal}}$ is obtained. Finally, the optimized VE-BRB model is established for diagnostic inference.

**Fig 3:**
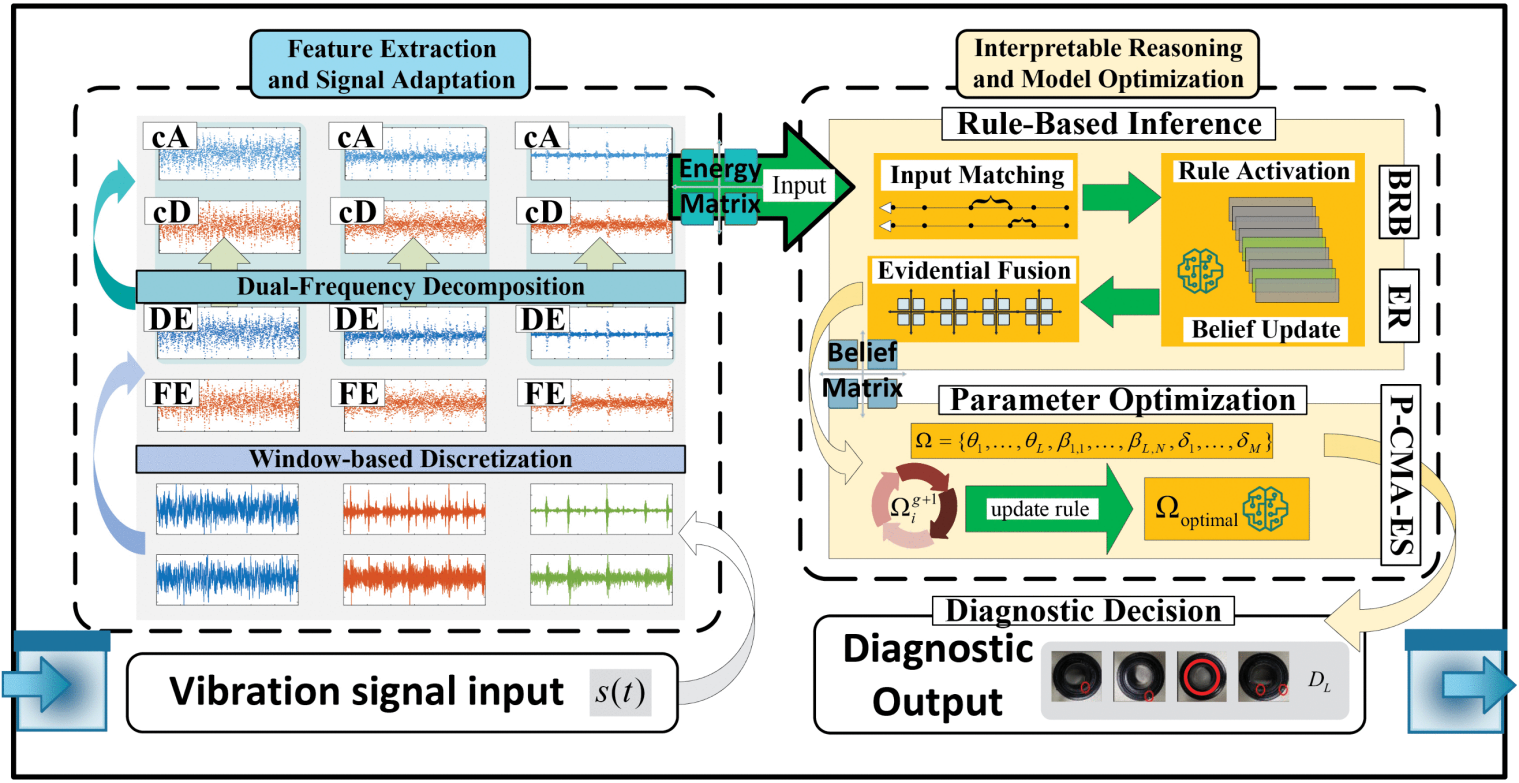
The overall framework of the VE-BRB model.

The computational complexity of the P-CMA-ES optimization primarily depends on the population size λ and the number of model parameters n, approximately scaling as $O\left(\lambda n^{\text{2}}\right)$ due to covariance matrix updates and offspring sampling. Although this increases the computational cost compared with gradient-based optimization, the process is executed offline during the model training phase, which are computationally lightweight. Therefore, VE-BRB remains feasible for online fault diagnosis. The framework of the proposed VE-BRB is shown in Fig 3.

## 4. Case study

This experiment utilized datasets from both the Case Western Reserve University (CWRU) and the Huazhong University of Science and Technology (HUST) bearing test platforms to illustrate the effectiveness of the VE-BRB.

### 4.1 Experimental setup and conditions

**Fig 4:**
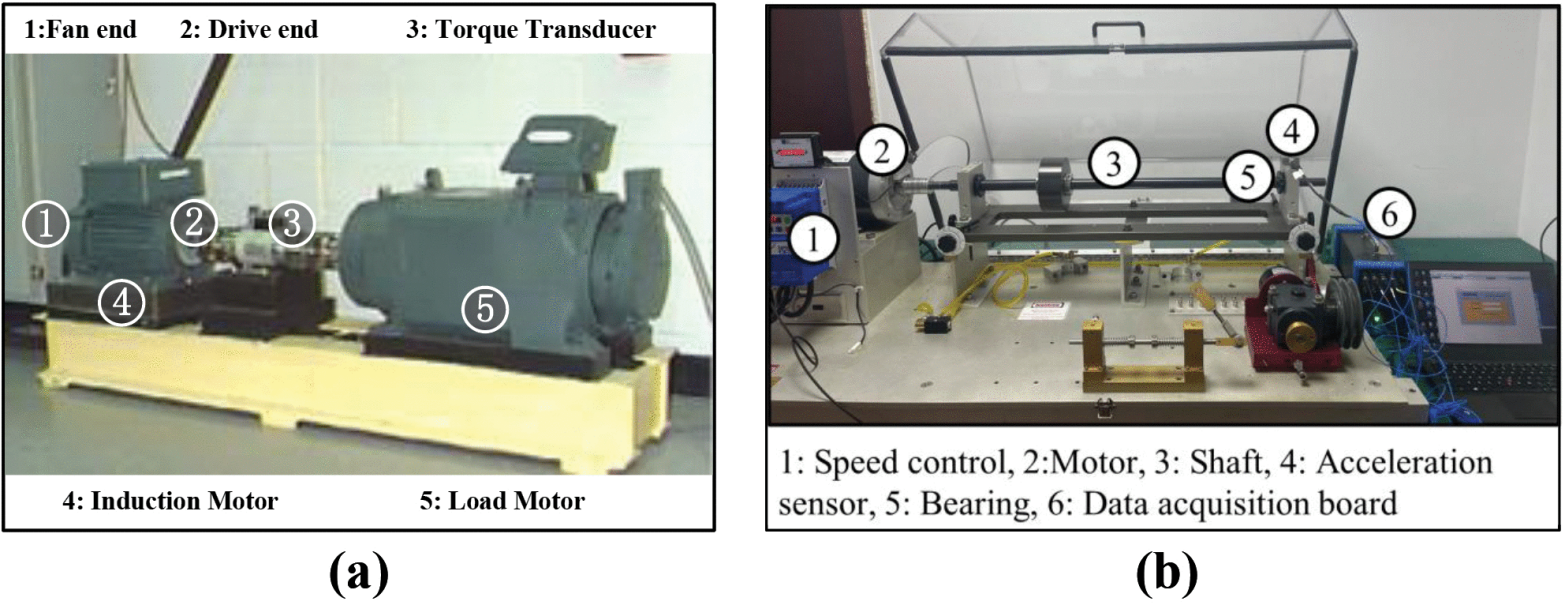
Dataset acquisition test rig. **(a)** From CWRU **(b)** From HUST.

The configurations and sensor placements of the CWRU and HUST bearing test rigs are depicted in Fig 4.

Case 1: The dataset was obtained from the CWRU bearing test platform, which consists of a 4 HP induction motor, rolling bearings, and a mechanical load system. Vibration signals were captured by accelerometers installed at the drive end (DE) and fan end (FE) of the motor housing. This study focused on three bearing states: normal (NO), inner-race fault (IR), and outer-race fault (OR). Each fault type was manufactured via electrical discharge machining (EDM) with diameters of 0.007 inches, 0.014 inches, and 0.021 inches. The signals were sampled at 12 kHz under a motor speed of 1730 rpm. Each fault sample contained 500 points (~1.2 revolutions).

Case 2: The dataset came from the HUST bearing test platform operating at 65 Hz. It includes combination fault (C) and three single fault types: rolling element (RE), inner race (IR), and outer race (OR). Signals were recorded at 25.6 kHz, with each fault sample containing 500 points (~1.27 revolutions). Compared with CWRU, the HUST dataset introduces more noise and diverse fault severities, providing a broader assessment of the model’s robustness and generalization.

**Fig 5:**
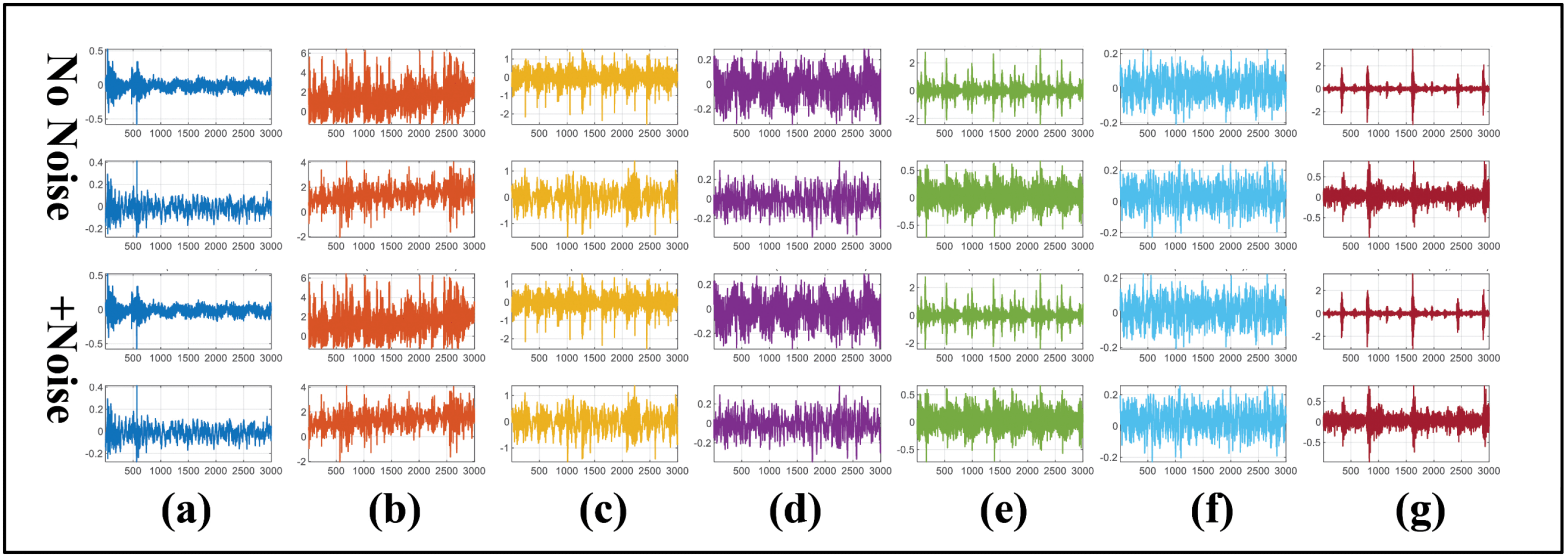
The X/Y and DE/FE acceleration signals from the HUST and CWRU datasets. **(a)** HUST-RE, **(b)** HUST-C, **(c)** HUST-IR, **(d)** HUST-OR, **(e)** CWRU-IR, **(f)** CWRU-NO, **(g)** CWRU-OR.

Fig 5 illustrates the acceleration signals of the bearing under different locations and noise conditions.

### 4.2 Experimental procedure

#### 4.2.1 Parameter setting.

Table 1 illustrates the reference input values used in the HUST dataset experiments, serving as an example for parameter configuration.

**Table 1:** Reference values for input signals.

Bearing State	IR	IR	OR	RE	RE
cD_X	3.543e + 01	1.257e-01	1.023e-02	8.795e-03	3.306e-14
cA_X	1.728e + 00	6.702e-03	1.358e-03	1.852e-04	3.026e-14
cD_Y	5.627e + 00	3.112e-04	1.642e-04	1.534e-04	1.314e-14
cA_Y	1.526e + 01	8.612e-03	3.623e-04	1.243e-04	2.423e-14

**Fig 6:**
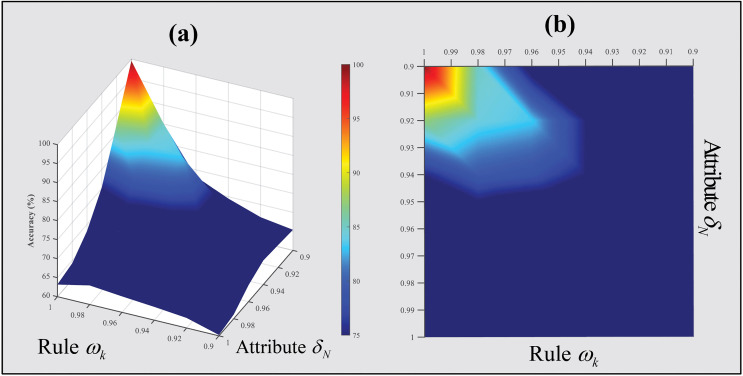
Impact of the initial values of rule weights and attribute weights on model accuracy.

The initial values of the rule weights (${\text{Rule W}}_{k}$) and attribute weights ($\text{Attribute}\delta_{N}$) were set to 1. Fig 6 shows the relationship among the initial values of rule weights, attribute weights, and model accuracy.

#### 4.2.2 Analysis of experimental results.

To further illustrate the interpretability and practical diagnostic capability of the proposed VE-BRB model, an example is conducted using a combination fault (C) bearing that simultaneously contains both IR and OR damages.

**Fig 7:**
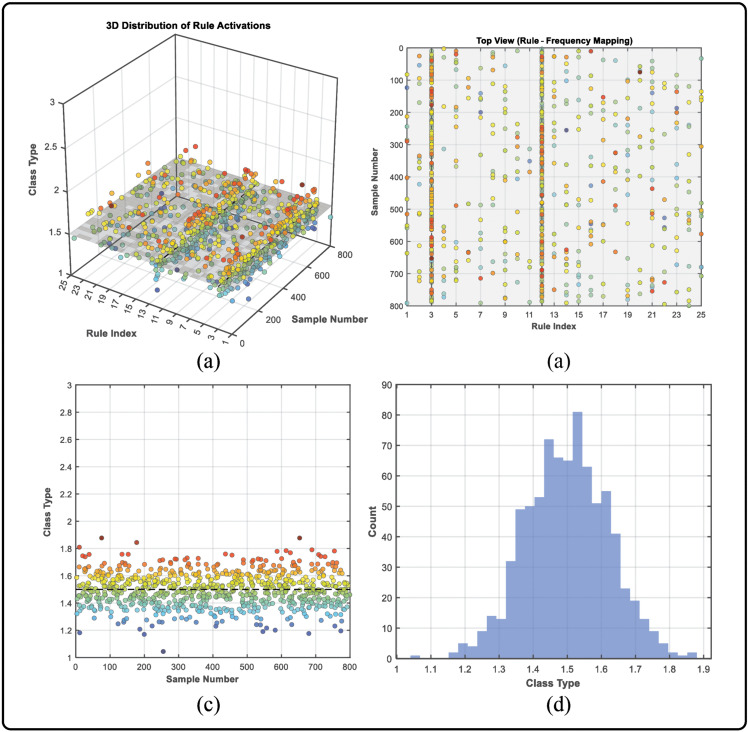
Diagnostic interpretation results of the proposed VE-BRB model under the combination fault (C) condition. **(a)** 3D visualization of rule activations; **(b)** Rule activation map based on the dual-frequency feature inputs; **(c)** Scatter distribution of the activation strength; **(d)** Histogram of the belief degree outputs corresponding to mixed fault states.

Fig 7 illustrates the diagnostic results for the bearing with the combination fault (C), which simultaneously contains inner-race (IR) and outer-race (OR) defects.

As shown in Fig 7(a)–7(c), the rule activations corresponding to the inner-race and outer-race conditions are both prominently excited.

In the 3D distribution, it can be observed that a large number of samples are concentrated between the IR (Class 1) and OR (Class 2) states, yet not closely attached to either class. This distribution indicates that these samples exhibit characteristics of both faults.

By analyzing the rule-activation diagram, users can trace the reasoning process through the dual-frequency features based on their respective physical interpretations, thereby identifying the underlying physical causes of the vibration anomalies.

The stable activation distribution around Class Type = 1.5 reveals the coexistence of IR and OR, while the belief degree outputs provide a quantitative measure of diagnostic confidence and reliability.

A total of 15,000 test samples were evaluated. The proposed VE-BRB model successfully predicted 14,928 samples on the CWRU dataset, achieving an overall accuracy of 99.52%, and 14,910 samples on the HUST dataset, with an accuracy of 99.40%. Under noisy conditions, accuracy slightly decreased to 98.84% on the CWRU dataset and 99.12% on the HUST dataset.

**Fig 8:**
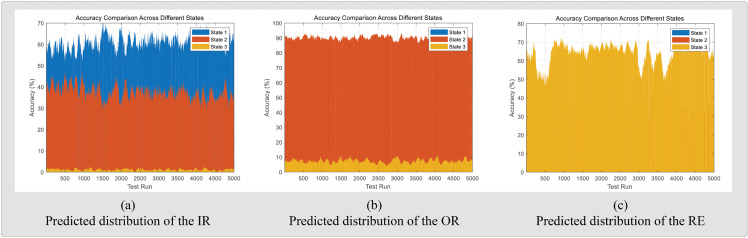
Distributions of predicted bearing states.

**Fig 9:**
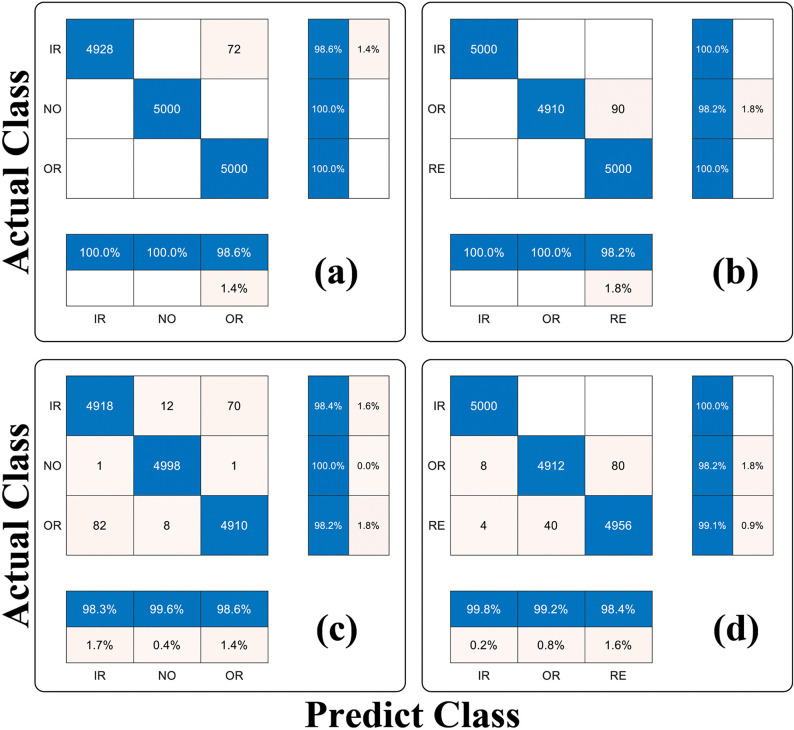
Confusion matrix for classification of bearing conditions.

Fig 8 illustrates the distribution of three diagnostic results obtained from the HUST dataset. Fig 9 presents the corresponding confusion matrices for both datasets under noise-free and noisy condition

#### 4.2.3 Comparative experiments.

To validate the performance and interpretability of the proposed VE-BRB model, six representative diagnostic methods were selected for comparison, covering traditional, deep, and knowledge-driven paradigms.

These include the S3VM, Physics-Informed Neural Network (PINN), Dual Cross-Attention Graph Convolutional Network (DCAGGCN), Adaptive Frequency Attention-based Interpretable Transformer (AFA-Transformer), and Target-Feature Enhancement with Feature-Boundary Alignment (TFE-FBA). In addition to conventional BRB, for all vector-based models, the feature datasets are first converted into vectorized representations. From each fault state of the two datasets, 10 and 20 samples are randomly selected as the training sets, while the remaining samples are used for testing. In addition, 1,000 samples are randomly labeled to support the semi-supervised training process. The accuracies of different methods are compared in Table 2.

**Table 2:** Diagnostic results of Case 1 and Case 2 under different conditions.

Method	Diagnostic Accuracy (%) under Different Conditions			
	Case 1	Case 1	Case 2	Case 2
	Noise-free	Noisy	Noise-free	Noisy
VE-BRB	99.52	98.84	99.40	99.12
S3VM	91.66	84.54	89.84	82.38
PINN	94.73	89.64	94.16	87.92
DCAGGCN	98.44	95.85	97.91	95.02
AFA-Transformer	99.35	98.51	99.16	98.44
TFE-FBA	98.96	97.99	98.69	97.53

As shown in Table 2, the VE-BRB model consistently attains the highest accuracy across all conditions (exceeding 99% under noise-free and remaining above 98.8% under noise). The S3VM achieves acceptable accuracy under noise-free conditions (around 91%) due to the separability of handcrafted statistical features, yet its performance drops markedly under noise owing to the lack of adaptability to nonlinear spectral variations. The Physics-informed Neural Network (PINN) maintains physically consistent predictions through embedded constraints, reaching approximately 94% in clean conditions, but its rigid formulation limits flexibility and thus leads to moderate performance (around 88%) when noise is present. The DCAGGCN performs well in clean environments with accuracy exceeding 98% by modeling inter-sensor correlations via graph-structured propagation, whereas its reliance on a stable topology causes a noticeable degradation (to about 95%) once the graph structure is perturbed by noise. The AFA-Transformer achieves excellent performance in both datasets (≈99%) by adaptively emphasizing fault-related frequency bands through its frequency-attention mechanism. The TFE-FBA effectively reduces domain discrepancy through target-feature enhancement and feature-boundary alignment, maintaining high generalization accuracy (around 99%) with only a minor decrease in noisy scenarios. These findings collectively verify that the proposed VE-BRB achieves an optimal balance between accuracy, interpretability, and robustness compared with both data-driven and physics-informed counterparts.

#### 4.2.4 Ablation experiment.

To further evaluate the contribution of vibration enhancement, an ablation study is conducted:

1. In Model #1, the window-based signal discretization is removed, and the input features are converted back into discrete reference levels as in the conventional BRB. This configuration is used to examine the role of continuous mapping in preserving the detailed vibration information.
2. In Model #2, the dual-frequency feature extraction module is removed, and the BRB receives only a single-band vibration feature as input. This setting is designed to verify the effectiveness of the proposed dual-frequency mechanism in capturing complementary fault characteristics.

**Fig 10:**
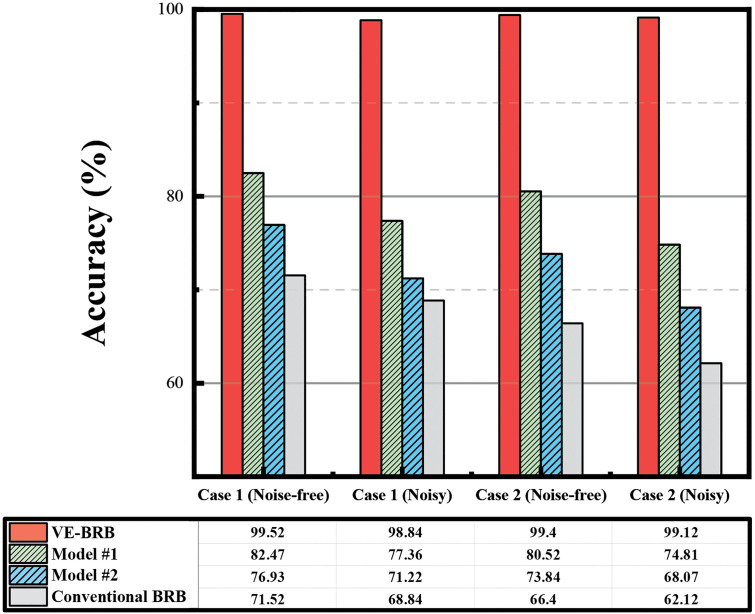
Ablation study results for VE-BRB.

Table 3 presents a detailed description of the ablation experiment. The average diagnosis results are shown in Fig 10.

**Table 3:** Detailed description of the ablation experiment.

Method	Window-Based Signal Discretization	Dual-Frequency Feature Extraction
VE-BRB	✓	✓
Model #1		✓
Model #2	✓	
Conventional BRB		

As shown in Fig 10, the proposed VE-BRB model consistently achieves the highest diagnostic accuracy under all test conditions. In contrast, Model #1 and Model #2 exhibit significant performance degradation once the vibration-based enhancement is partially removed.

For Model #1, the removal of the window-based discretization forces the vibration input to revert to conventional discrete reference. This modification prevents the BRB from learning wave-frequency information in continuous input signals, resulting in the loss of subtle temporal patterns representing transitional or compound fault behaviors. Consequently, the diagnostic capability of Model #1 deteriorates markedly.

For Model #2, the removal of the dual-frequency feature extraction module prevents the model from capturing the complementary diagnostic cues embedded in both low- and high-frequency bands. Without this dual-frequency representation, Model #2 loses sensitivity to one of these essential signal aspects, resulting in incomplete fault feature representation and reduced discriminative ability.

The conventional BRB, which lacks both mechanisms, shows the lowest accuracy and exhibits severe sensitivity to noise due to discretization errors and rule sparsity. The proposed vibration-based enhancement allows the BRB model to directly process continuous vibration signals without discretization loss. Compared with the conventional BRB, it is therefore more suitable for handling raw signal inputs and capturing detailed fault-related information.

## 5. Conclusions and outlook

This study proposed a novel Vibration-Enhanced Belief Rule Base (VE-BRB) model for interpretable bearing fault diagnosis. By introducing a window-based discretization mechanism and a dual-frequency feature extraction strategy, the model enables BRB reasoning to directly process continuous vibration signals while preserving the physical interpretability of low- and high-frequency components.

The results from both CWRU and HUST datasets demonstrate that VE-BRB achieves high diagnostic accuracy and strong robustness under noisy and small-sample conditions, while maintaining transparent, rule traceability throughout the inference process. Compared with black-box models, the proposed method establishes a clearer causal link between vibration responses and diagnostic outcomes, thereby offering an interpretable alternative for safety-critical applications.

Several important research directions remain open for exploration. Future work will focus on the real-time deployment of the VE-BRB framework in industrial monitoring systems, emphasizing lightweight implementation and computational efficiency for online decision-making. Furthermore, rule simplification and automatic pruning mechanisms will be explored to reduce model redundancy and improve scalability, facilitating large-scale industrial adoption. These developments will advance the VE-BRB from a research-oriented framework toward a practical, deployable solution for interpretable and reliable fault diagnosis in complex machinery systems.

## References

[1] Liu Z, Zhang L. A review of failure modes, condition monitoring and fault diagnosis methods for large-scale wind turbine bearings. Measurement. 2020;149:107002. doi: 10.1016/j.measurement.2019.107002

[2] Wang H, Liu Z, Peng D, Zuo MJ. Interpretable convolutional neural network with multilayer wavelet for Noise-Robust Machinery fault diagnosis. MSSP. 2023;195:110314. doi: 10.1016/j.ymssp.2023.110314

[3] Sjöberg J, Zhang Q, Ljung L, Benveniste A, Delyon B, Glorennec P-Y, et al. Nonlinear black-box modeling in system identification: a unified overview. Automatica. 1995;31(12):1691–724. doi: 10.1016/0005-1098(95)00120-8

[4] Chen Q, Zhang F, Wang Y, Yu Q, Lang G, Zeng L. Bearing fault diagnosis based on efficient cross space multiscale CNN transformer parallelism. Sci Rep. 2025;15(1):12344. doi: 10.1038/s41598-025-95895-x 40210923 PMC11985507

[5] Bai H, Tong W, Geng Z, Gao C. A rolling bearing fault diagnosis method based on an improved parallel one-dimensional convolutional neural network. PLoS One. 2025;20(8):e0327206. doi: 10.1371/journal.pone.0327206 40788891 PMC12338813

[6] Song X, Lyu X, Sun S, Li C. A novel deep learning model for fault diagnosis of rolling-element bearing based on convolution neural network and recurrent neural network. Proc Inst Mech Eng Part E: J Process Mech Eng. 2023;239(4):1803–13. doi: 10.1177/09544089231191042

[7] Sahu D, Dewangan RK, Matharu SPSJ. Long short-term memory based fault diagnosis of rolling element bearings using vibration signals. Control. 2025:10775463251328176.

[8] Serban A, van der Blom K, Hoos H, Visser J. Practices for engineering trustworthy machine learning applications. 2021 IEEE/ACM 1st Workshop on AI Engineering-Software Engineering for AI (WAIN). IEEE. 2021. p. 97–100.

[9] Verenich I, Nguyen H, La Rosa M, Dumas M. White-box prediction of process performance indicators via flow analysis. Proceedings of the 2017 International Conference on Software and System Process; 2017. p. 85–94. doi: 10.1145/3084100.3084110

[10] Wang X, Chen YJSS. Application of fault tree analysis in ship cargo crane hydraulic system. Technology. 2023;45(3):186–9.

[11] Jørgensen SB, Hangos KM. Grey box modelling for control: qualitative models as a unifying framework. Adapt Control Signal. 1995;9(6):547–62. doi: 10.1002/acs.4480090607

[12] Gacto MJ, Alcalá R, Herrera F. Interpretability of linguistic fuzzy rule-based systems: an overview of interpretability measures. Inf Sci. 2011;181(20):4340–60. doi: 10.1016/j.ins.2011.02.021

[13] Cao Y, Zhou Z, Hu C, He W, Tang S. On the interpretability of belief rule-based expert systems. IEEE Trans Fuzzy Syst. 2021;29(11):3489–503. doi: 10.1109/tfuzz.2020.3024024

[14] Soni R, Mehta B. Diagnosis and prognosis of incipient faults and insulation status for asset management of power transformer using fuzzy logic controller & fuzzy clustering means. Electr Power Syst Res. 2023;220:109256. doi: 10.1016/j.epsr.2023.109256

[15] Feng L, Ding Z, Yin Y, Wang Y, Zhang Q, Liu X, et al. Scraper conveyor gearbox fault diagnosis based on multi-source heterogeneous data fusion. Measurement. 2025;247:116797. doi: 10.1016/j.measurement.2025.116797

[16] Siddique MF, Saleem F, Umar M, Kim CH, Kim J-M. A hybrid deep learning approach for bearing fault diagnosis using continuous wavelet transform and attention-enhanced spatiotemporal feature extraction. Sensors (Basel). 2025;25(9):2712. doi: 10.3390/s25092712 40363151 PMC12074297

[17] Zhou H, Liu R, Li Y, Wang J, Xie S. A rolling bearing fault diagnosis method based on a convolutional neural network with frequency attention mechanism. Struct Health Monit. 2023;23(4):2475–95. doi: 10.1177/14759217231202543

[18] Li Y, Zhang X, Chen Z, Yang Y, Geng C, Zuo MJ. Time-frequency ridge estimation: an effective tool for gear and bearing fault diagnosis at time-varying speeds. MSSP. 2023;189:110108. doi: 10.1016/j.ymssp.2023.110108

[19] Feng Z, He W, Zhou Z, Ban X, Hu C, Han X. A new safety assessment method based on belief rule base with attribute reliability. IEEE/CAA J Autom Sinica. 2021;8(11):1774–85. doi: 10.1109/jas.2020.1003399

[20] Zhichao M, Zhijie Z, You CAO, Shuaiwen T, Yuan C, Xiaoxia HAN, et al. A new interpretable fault diagnosis method based on belief rule base and probability table. Chin J Aeronaut. 2023;36(3):184–201. doi: 10.1016/j.cja.2022.08.003

[21] Xiao F. GEJS: a generalized evidential divergence measure for multisource information fusion. IEEE Trans Syst Man Cybern Syst. 2023;53(4):2246–58. doi: 10.1109/tsmc.2022.3211498

[22] Zhi S, Su K, Yu J, Li X, Shen HJSHM. An unsupervised transfer learning bearing fault diagnosis method based on multi-channel calibrated Transformer with shiftable window. 2025:14759217251324671.

[23] Yuan L, Wang H. Application of hierarchical symbolic fuzzy entropy and sparse Bayesian ELM to bearing fault diagnosis. J Mech Sci Technol. 2023;37(5):2241–52. doi: 10.1007/s12206-023-0401-1

[24] Wu Y, Li C, Yang S, Bai Y. Multiscale reduction clustering of vibration signals for unsupervised diagnosis of machine faults. Appl Soft Comput. 2023;142:110358. doi: 10.1016/j.asoc.2023.110358

[25] Chen L-Y, Zhou Z-J, Zhang C-C, Li C, Chen M-L. A performance evaluation method based on interval evidential reasoning approach with function monotonicity. IEEE Trans Instrum Meas. 2022;71:1–15. doi: 10.1109/tim.2022.3219487

[26] Zhou Z, Chen L, Han X, Tang S, Li G. An interval evidential reasoning-based dynamic performance evaluation method for complex systems. Comput Ind Eng. 2021;162:107735. doi: 10.1016/j.cie.2021.107735

[27] Kim I, Wook Kim S, Kim J, Huh H, Jeong I, Choi T, et al. Single domain generalizable and physically interpretable bearing fault diagnosis for unseen working conditions. Expert Syst Appl. 2024;241:122455. doi: 10.1016/j.eswa.2023.122455

[28] Zhou Z-J, Hu G-Y, Zhang B-C, Hu C-H, Zhou Z-G, Qiao P-L. A model for hidden behavior prediction of complex systems based on belief rule base and power set. IEEE Trans Syst Man Cybern Syst. 2018;48(9):1649–55. doi: 10.1109/tsmc.2017.2665880

[29] Yang J-B, Liu J, Wang J, Sii H-S, Wang H-W. Belief rule-base inference methodology using the evidential reasoning approach-RIMER. IEEE Trans Syst Man Cybern A. 2006;36(2):266–85. doi: 10.1109/tsmca.2005.851270

[30] Wang Y, Liu H, Jia W, Guan S, Liu X, Duan X. Deep fuzzy rule-based classification system with improved wang–mendel method. IEEE Trans Fuzzy Syst. 2022;30(8):2957–70. doi: 10.1109/tfuzz.2021.3098339

[31] Zhang X, Chung F-L, Wang S. An interpretable fuzzy DBN-based classifier for indoor user movement prediction in ambient assisted living applications. IEEE Trans Ind Inf. 2020;16(1):42–53. doi: 10.1109/tii.2019.2912625

